# Lornoxicam controlled release transdermal gel patch: Design, characterization and optimization using co-solvents as penetration enhancers

**DOI:** 10.1371/journal.pone.0228908

**Published:** 2020-02-27

**Authors:** Durriya Hashmat, Muhammad Harris Shoaib, Fatima Ramzan Ali, Fahad Siddiqui

**Affiliations:** Department of Pharmaceutics, Faculty of Pharmacy and Pharmaceutical Sciences, University of Karachi, Karachi, Pakistan; University of New South Wales, AUSTRALIA

## Abstract

The aim of the current study was to develop membrane-based transdermal patches of lornoxicam gel using oleic acid (OA)and propylene glycol (PG) as penetration enhancers to improve drug delivery across the skin and to evaluate in vivo analgesic and anti-inflammatory activity. For this purpose, nine formulations were developed in accordance with 3^2^ factorial design using Design Expert^®^ 11. The concentration of propylene glycol (*X*_1_) and oleic acid (*X*_2_) were selected as independent variable whereas Q_10_ (*Y*_1_), flux (*Y*_2_) and lag time (*Y*_3_) were considered as the response variables. The impact of drug loading, surface area, gel concentration, membrane variation and agitation speed on drug release and permeation was also studied. The skin sensitivity reaction, analgesic activity and anti-inflammatory action of the optimized patch were also determined in Albino Wistar rats. Stability studies were performed for three months at three different temperature conditions. The result suggests that a membrane-based system with controlled zero-order drug release of 95.8 ± 1.121% for 10 h exhibiting flux of 126.51±1.19 μg/cm^2^/h and lag time of 0.908 ±0.57h was optimized with the desired analgesic and anti-inflammatory effect can be obtained by using propylene glycol and oleic acid co-solvents as a penetration enhancer. The patch was also found stable at 4˚C for a period of 6.44 months. Formulation F9 comprising of 10% PG and 3% OA was selected as an optimized formulation. The study demonstrates that the fabricated transdermal system of lornoxicam can deliver the drug through the skin in a controlled manner with desired analgesic and anti-inflammatory activity and can be considered as a suitable alternative of the oral route.

## Introduction

The nonsteroidal Anti-inflammatory Drugs (NSAIDS) have been extensively recommended for the treatment of inflammatory disorders including osteoarthritis and Rheumatoid arthritis. Oral administration of NSAIDS exhibits efficient relief in inflammatory diseases. However, the adverse effects such as irritation and ulceration of gastro-intestinal mucosa have limited the clinical use of NSAIDs [1]. One of the approaches to avoid toxicity and using NSAIDS for a longer period of time is its administration via transdermal route.[2]

Transdermal systems by delivering drugs across the skin into systemic circulation resist the alteration in absorption rate, metabolism and prevent the gastrointestinal adverse effects occurring due to oral administration of drugs.[3, 4] It is ideally suited for chronic disorders. [5] It allows the administration of potent drugs with the benefit of self–administration and enhanced therapeutic efficacy.[6]

Lornoxicam (LRX) is a potent NSAID of oxicam class. It is extensively prescribed in chronic painful and inflammatory conditions. Like other NSAIDS, oral administration of LRX leads to numerous gastrointestinal, renal and hematological adverse effects.[7]. Apart from these, it needs frequent administration due to its short half-life (3-4h). Moreover, parenteral administration is not recommended against chronic conditions[8]

The controlled delivery of LRX through the transdermal route is an advantageous approach to overcome all the concerns associated with LRX administration. In the present study, LRX has been formulated as reservoir transdermal patches. The reservoir transdermal patches contain the drug in a separate compartment which can be delivered by a simple diffusion process providing controlled drug delivery over a required period of time. Since it follows zero order kinetics, there is an effective control on release rate.

The skin acts as an excellent barrier to the transdermal permeation of drugs. Different chemical enhancers have been introduced in order to enhance transdermal drug delivery. These chemical enhancers by interacting with the skin constituents improve the flux of drug molecules [9]. These agents reversibly alter the skin’s barrier function and subsequently allow the molecules with poor penetration to surpass stratum corneum and ultimately enter the systemic circulation.[10] Numerous studies have shown that penetration enhancers, when used in combination can produce synergistic action thereby promoting skin permeation greater than individual penetration enhancers. [11]. Co-solvents have been extensively used as vehicles and penetration enhancers in transdermal formulations. These agents not only enhance the drug solubility but also alter the structure of the skin and hence improve the penetration rate. Thus, affecting both drug release and permeation.[12]

Many fatty acids have been used to improve percutaneous drug absorption including oleic acid (OA). The use of OA with propylene glycol (PG) proves to be a very successful combination in many transdermal formulations.[9, 13, 14]

Hence, the aim of the current study is to improve the transdermal permeation of LRX by using PG and OA co-solvent as a penetration enhancer. For this purpose, reservoir type transdermal patches of LRX has been developed using a rate-controlling membrane, which can control the release at a constant rate for a period of 10 h.

## 2. Materials and methods

### 2.1. Materials

Lornoxicam was gifted by ATCO Laboratories Ltd., Pakistan. Other chemicals/excipient used were Carbopol 940, oleic acid (OA) and Propylene Glycol (PG) (Dae-Jung, Gyeonggi-do, Korea), Triethanolamine (BDH, Poole, England) and Methanol HPLC grade (TEDIA, Fairfield, USA). The backing membrane (3M-9720), rate-controlling membranes (3M Cotran-9728 and 3M CoTran-9716) and release liner (SCOTCHPAK—9755) were gifted by 3M, St. Paul, USA. Acrylate adhesive Duro–Tak 387/2510 was supplied by Henkel Corporation (Bridgewater, USA).

### 2.2. Animals

The animals used for skin irritation and analgesic/anti-inflammatory studies were Male Wistar Albino Rats (weighing 150–180 g) provided by the animal house, Faculty of Pharmacy and Pharmaceutical Sciences, University of Karachi, Pakistan. All ARRIVE guidelines for the maintenance and utilization of laboratory animals were followed. The animals were kept under standard laboratory conditions with free access to food and water. All the protocols followed for animal studies were reviewed and approved by the Institutional Bioethics Committee, University of Karachi (IBC KU 51).

### 2.3. Solubility studies of lornoxicam

Excess quantity of LRX powder was taken in separate conical flasks containing 10 mL phosphate buffer saline (PBS) pH 5.8, pH 6.8, pH 7.4 and water. The flasks were continuously stirred for 72h using orbital shaker bath (Model SW-23, Julabo, USA). An aliquot was withdrawn and filtered using Millipore filter 0.45 μm. The dilution was analyzed spectrophotometrically at 376 nm. The concentration was determined in triplicate[15].

### 2.4. Experimental design

A 3^2^ full factorial design was used to design the experiments using Design-Expert version 11 (Stat-ease Inc, Minneapolis, USA). PG (*X*_1_) and OA (*X*_2_) were selected as independent variables whereas drug release at 10h, Q_10_ (*Y*_1_), flux (*Y*_2_) and lag time (*Y*_3_) were considered as dependent response variables. Nine formulations of gel were constructed as specified in Table 1. For each response, the polynomial equation was generated and from the results obtained, the optimized formulation was selected.

**Table 1:** Composition of lornoxicam gel for membrane-based transdermal patches.

Ingredients	Formulation Code								
(% w/w)	F1	F2	F3	F4	F5	F6	F7	F8	F9
Lornoxicam	0.8	0.8	0.8	0.8	0.8	0.8	0.8	0.8	0.8
Propylene glycol (PG)	15	5	15	5	10	5	10	15	10
Oleic acid (OA)	1	5	5	1	5	3	1	3	3
Methyl paraben	0.2	0.2	0.2	0.2	0.2	0.2	0.2	0.2	0.2
Triethanolamine	2	2	2	2	2	2	2	2	2
Carbopol (940)	0.5	0.5	0.5	0.5	0.5	0.5	0.5	0.5	0.5
Distilled water	100	100	100	100	100	100	100	100	100

### 2.5. Formulation of lornoxicam gel

For the preparation of gel, carbopol was soaked in water overnight. Then, a measured quantity of LRX was dissolved in triethanolamine. Methylparaben, PG, OA and 10 mL of water were dissolved in the drug mixture with constant stirring. The solvent blend was mixed with carbopol and agitated for additional 20 minutes until a homogeneous gel was formed.[16]

### 2.6. Physicochemical characterization of lornoxicam gel

The prepared LRX gels were assessed for color, homogeneity, viscosity, pH and drug content. The pH was measured using a calibrated pH meter (Mettler MP-220, Switzerland). Brookfield viscometer (Model DV II, USA) was used to determine the viscosity of gel at 25˚C; the spindle was set at 20 rpm. Each test was repeated in triplicate.

### 2.7. Dose calculation for transdermal formulation

The dose calculation for controlled drug delivery has been illustrated by Baker [17]which is given as:

$$t_{e}=t_{1/2}\frac{Mo-Me}{ln2Me}$$ (1)

Where, M_0_ is a required dose of the drug, t_e_ is the required duration of action, M_e_ is Minimum effective concentration and t_1/2_ is the half-life of the drug.

LRX has a minimum effective concentration of 1 mg/L with a half-life of 4h and volume of distribution of 14 L.[18]. The calculated dose used in the formulated patches was 38.22mg.

### 2.8. Fabrication of membrane-based lornoxicam patches

The reservoir-type TDS of LRX was fabricated by sandwiching the reservoir gel between the backing layer (3M -9720) and an ethylene-vinyl acetate (EVA) membrane (3M Cotran– 9728). First, the EVA membrane and the backing layer was heat sealed and cut to an appropriate size (30 cm^2^). Then, accurately weighed quantity of gel (5 g) was filled in the device by means of a disposable syringe. The device was heat-sealed again ensuring no leakage of the reservoir gel from the device. A pressure sensitive adhesive polymer (Duro-tak 387/2510) was coated onto the EVA membrane to ensure intimate contact of the patch to the skin. Finally, a release liner (Scotchpak—9755) was pressed over the adhesive coated rate-controlling membrane. The patches were stored in aluminum foil at room temperature.[19]

### 2.9. Characterization of fabricated patches

#### 2.9.1. Weight

Three patches from each formulation were randomly selected and weighed individually. Then, the average weight and standard deviation were calculated. [20]

#### 2.9.2. Thickness

The thickness of the patches was measured by means of a micrometer screw gauge at the 4 edges and the center of the patch. The results were expressed as average thickness ± SD. **[21]**

#### 2.9.3. Content uniformity of patches

*2*.*9*.*3*.*1*. *Sample preparation*. The reservoir compartment containing drug was extracted with 100 mL of PBS pH 7.4. The flask was stirred for 4 h using a mechanical shaker (IKA, KS 260 basic, Germany). The solution was filtered, and drug content was determined by the HPLC method.

*2*.*9*.*3*.*2*. *HPLC analysis*. The quantification of LRX was performed using the HPLC method described by Gonullu et al with minor modifications[7]. The HPLC unit was equipped with pump (Shimadzu 20AD), UV detector (Shimadzu SPDM -20A, photo diode array (PDA) and C-18 column (250 × 4.6 mm, 5 μm particle size, Mediterranean Sea®, Teknokroma, Barcelona, Spain). The samples were detected at 376 nm and data acquisition was performed using software (Lab solutions, Version 6.72 SP 1). The mobile phase consisted of aqueous phosphate buffer and methanol in a ratio of 6:4 v/v having a pH of 7 adjusted by 1 M sodium hydroxide. Its flow rate was 1 mL/min and the volume of sample injected was 20 μL. The LOD and LOQ values were 0.1 and 0.5 μg/mL, respectively.

A standard calibration curve of LRX was constructed in the range of 0.5–20 μg/mL. To evaluate the linearity, drug determination was carried out in the mobile phase. Good linearity was observed between the concentration of lornoxicam and the peak area of lornoxicam with a high correlation coefficient (r^2^ = 0.999). The standard curve constructed was used for estimating drug content in lornoxicam patches.

#### 2.9.4. In vitro release study

The in vitro release profile of LRX from reservoir patches was determined by using USP Apparatus V (Paddle-over disk apparatus) (Erweka DT-600, Heusenstamm, Germany). The vessels were filled with 500 mL of PBS pH 7.4 and the temperature was maintained at 32 ± 0.2˚C. The fabricated reservoir patches (30 cm^2^) were sandwiched between a watch glass and a wire-screen (17-inch mesh) (Labecx, Santa Clarita California, USA) and immersed into the dissolution medium. Aliquots of 5 ml were withdrawn and replaced with fresh medium at specified time points i.e. 0.5, 1, 2, 3, 4,5, 6, 7, 8, 9 and 10 h. The samples were suitably diluted and analyzed by means of a double beam UV-Spectrophotometer at 376 nm [22]. Each experiment was repeated in triplicate.

Various kinetic models were used to determine the release kinetics such as zero-order, first-order, Higuchi model Korsmeyer—Peppas model, Weibull and Makoid–Banakar model [23, 24]. The coefficient of correlation (R^2^) and rate constants was determined using Excel Add-In Program DD Solver^®^ [25] and values of R^2^ were compared to determine the best-fitted model. The equations of models are as follows:

**Zero-order:**

$$Q_{t}=Q_{o}+k_{o}t$$ (2)

where Q_o_ and Q_t_ represent the initial amount of drug in dosage form and amount of drug at time t, respectively. k_𝑜_ is a zero-order rate constant.

First—order:

$$\log\;Q_{t}=\log\;Q_{o}+\frac{k_{1}t}{2.303}$$ (3)

Where k_1_ is the first order rate constant.

**Higuchi model:**

$$Q_{t}=k_{H}t^{1/2}$$ (4)

Where k_H_ is the Higuchi rate constant.

**Korsmeyer-Peppas model:**

$$\frac{M_{t}}{M_{\infty }}=Kt^{n}$$ (5)

Where, MtM0 is the fraction of the drug released at time t, *K* is rate constant and n is the release exponent indicating the drug release mechanism.

**Weibull model:**

$$m=1-exp\left[\frac{{-(t-T_{l})}^{b}}{a}\right]$$ (6)

Where ‘m’ is drug accumulated fraction in solution at any time t. The scale parameter is, a, defining time scale process. Lag time is presented by T_*l*_ i.e. the time required before the onset of drug release, in most cases, it will be zero. b is considered as a shape parameter and expresses curve.

**Makoid-Banakar model:**

$$\frac{M_{t}}{M_{\infty }}=k_{MB}t^{n}e^{\left(-ct\right)}$$ (7)

Where, K_MB_, n, and care empirical parameter (K_MB_, n, c > 0) and M_t_/M_∞_ is the accumulation fraction of the drug in solution at time t.

#### 2.9.5. In vitro permeation study

The skin of Wistar albino rats (150–180 g) was used for in vitro permeation studies. The rats were sacrificed by giving anesthesia (ether). The skin hair of animals was surgically removed with an electrical clipper. To remove adhering fat, the dermis side of the skin was wiped with isopropyl alcohol. After washing with PBS, the skin was enclosed in aluminum foil and stored in a deep freezer at -20˚C until further use. Before the experiment, the skin was brought to room temperature and placed carefully in the vessel containing the dissolution medium.[26]

For in vitro permeation studies, Franz diffusion cell is commonly used equipment. However, reservoir patches with larger surface area can’t be fitted into the donor chamber of its cell without cutting, which results in loss of patch integrity. Prodduturi et al. recommended the use of USP apparatus V for permeation studies of such patches. [27]Therefore, in the present study, USP apparatus V was used for permeation studies of LRX reservoir patches.

The vessel was filled with 600 mL of Hank’s balanced salt solution. The sample patch was applied on the rat skin which was then adjusted between watch glass and wire screen and placed in the vessel. The temperature was maintained at 32 ± 0.2˚C and the rotation of apparatus was adjusted at 100 rpm. Aliquots of 10 ml were withdrawn at 0.5, 1, 2, 3, 4, 5, 6, 7, 8, 9 and 10 h and were substituted with an equivalent volume of the medium. Each sample was diluted and analyzed using the HPLC mentioned above for determining the content uniformity of drug in patches [7]. Each experiment was performed in triplicate. The cumulative amount of drug permeated through rat skin was plotted against time for all the formulated patches. [28]

Flux (J, μg/cm^2^/h) was determined from the slope of the linear portion of cumulative amount permeated per unit area vs time, and time lag (T_L_) was determined by extrapolation of the line to the abscissa.[29]. The permeability coefficient (P) was calculated using Fick’s First Law of diffusion which is described as

$$P=J/C_{o}$$ (8)

Where *J* is the steady-state permeation flux and *C*_*o*_ is the initial concentration.

The diffusion coefficient (DC) was calculated using the relation derived from Fick’s second law of diffusion which is described in Eq 9:

$$DC=\frac{h^{2}}{6T_{l}}$$ (9)

Where T_*l*_ is the lag time and h is the thickness of the skin. [30]

The rate of drug delivery is either controlled by the device or stratum corneum. [31].The fraction rate controlled by the device (F_D_) and skin (F_S_) is computed by the following equations:

$$F_{D}=\frac{M_{total}}{M_{device}}$$ (10)

$$F_{S}=1-F_{D}$$ (11)

#### 2.9.6. Evaluation of formulation factors

The impact of varying formulation factors such as concentration of carbopol (0.5%, 1%, 1.5%), drug loading concentrations (20 mg, 30 mg, 40 mg), surface area (20 cm^2^, 30 cm^2^, 40 cm^2^), rate controlling membranes (3M Cotran-9728 and 3M CoTran-9716), adhesive DURO-TAK 387/2510 and different rpm (50, 75 and 100 rpm) on drug release and permeation of optimized transdermal reservoir patch was also investigated. For the evaluation of each factor, separate patches were developed as described previously.

### 2.10. Skin irritation study

The skin irritation studies were carried out according to the method described by Draize et al.[32] The animals were divided into three groups each containing six rats. Group I served as the control, group II was treated with optimized LRX patch while group III received 0.8% aqueous solution of formalin as the standard irritant. The rats were clipped free of hair 24 h before the experiment. The patches were applied on the rat skin and the application site was covered and wrapped with an elastic adhesive bandage. The skin reactions were evaluated in accordance with the Draize method. [33].

### 2.11 In vivo analgesic and anti-inflammatory activities

#### 2.11.1. Hot-plate reaction time

The analgesic activity of LRX was performed by the Hot-plate Analgesic method. This method was reported by Baker et al[34] and was adopted with slight modifications for reservoir patches. Animals were divided into three groups (n = 6 for each group). Group I served as control, group II served as standard and received 4mg/kg LRX orally [35] and group III was treated with the optimized patch (4 cm^2^). The test patch was applied topically on the posterior paw of each rat of the treated group and the response time was recorded after 0, 30, 60, 120 and 180 min after application. Each rat was placed on a hot plate (MOD 39D Hot meter, Columbus, USA) maintained at 55 ± 1˚C and the latency period was recorded. The responses to pain stimulus include jumping/raising and licking of the hind foot. The animals were observed for any gross behaviour changes, morbidity and mortality.

#### 2.11.2. Acetic acid-induced writhing response

Writhing induced by acetic acid was also used to assess the analgesic effect of LRX reservoir patch. Male Albino rats were divided into three groups(n = 6 for each group). The animals were weighed and numbered appropriately. Normal saline was given to the control group (Group I), the standard group (group II) received oral LRX (4 mg/kg) and the test group (group III) received an optimized LRX patch (4 cm^2^) prior to administration of acetic acid. The hair on the abdominal skin of rats was removed 12 h prior to the application of the patch. After 1 h of application, the patches were removed and acetic acid in the concentration of 1% was injected intraperitoneally to all groups. Five minutes later, the number of writhings (W) within 20 minutes was recorded. **[36]**The pain inhibition ratio (PIR) was calculated according to the following formula:

$$PIR=\frac{W_{blank}-W_{administration}}{W_{blank}}$$ (12)

### 2.11.3 Carrageenan hind paw edema test

The anti-inflammatory activity of LRX patches was evaluated through the Carrageenan-hind paw edema test reported by Winter et al in 1962[37]. The method was adopted with slight modifications. The animals were divided into three groups, each comprising of six animals. Group I served as control (only carrageenan is administered). Group II was treated with oral LRX (4 mg/kg). Group III was treated with the optimized patch (4 cm^2^) which was applied topically on the left hind paw. The test patch was applied 1 h prior to the carrageenan injection. After 1 h, 0.1 mL of carrageenan solution (1%) was injected in the left hind paw of all groups. The paw edema was measured at 1, 2, 3, 4, 5 and 6 h using a Vernier caliper (Seiko brand, China). The percent inhibition of edema was calculated through the following equation:

$$\%\;edema\;Inhibition=\frac{{\%edema}_{\left(control\right)}-{\%edema}_{\left(drug\right)}}{{\%edema}_{\left(control\right)}}\times 100$$ (13)

#### 2.11.4 Statistical analysis

For in vivo studies, the difference between control groups and treated groups was assessed by one-way ANOVA using Tukey’s multiple comparison post–hoc test. A probability level of P<0.05 was considered statistically significant. Statistical evaluations were performed by SPSS (Version 20).

### 2.12. Stability studies

Accelerated stability studies for the designed patches were performed by storing the replicates of LRX patches under three different temperature conditions i.e. at 4°C, room temperature and 45°C[38]. The samples were analyzed at an interval of 0, 30, 60 and 90 days for physical appearance and drug content determination. HPLC method was used for evaluating the drug content[7]. In vitro permeation study was also performed in triplicate for samples stored at 4°C.

## Result and discussion

### 3.1 Solubility of lornoxicam

The solubility of a drug plays an important role in obtaining appropriate bioavailability. The main hindrance which comes across in the development of new drug molecules is low aqueous solubility. Most of the drugs are either weakly acidic or weakly basic and have poor aqueous solubility. Lornoxicam is also one of those drugs which exhibit poor aqueous solubility. The solubility studies for the selected drug was carried out in the water, Phosphate buffer pH 5.8, 6.8 and 7.4. According to the results, least solubility was observed in water i.e. 0.038±0.001 mg/ml and the highest solubility was found in phosphate buffer pH 7.4 .i.e. 0.346±0.002mg/ml. These results were parallel with the findings of Mundada et al where the solubility was highest in phosphate buffer 7.4 and was found to be 0.303 ± 0.008mg/ml [2]

### 3.2 Characterization of gels

Table 2 represents the physicochemical characteristics of the formulated gels. All gels exhibited appropriate cosmetic qualities such as uniform color, homogeneity, smooth texture and no phase separation. The pH of gel formulations F1-F9 ranged between 6.25 ± 0.03 and 6.80 ± 0.045. The pH values were found closer to 7, which is suitable for transdermal preparation. [39]. The viscosity of the gels ranged between 1300 ± 0.83 cps and 5130 ± 0.59 cps and the drug content was found within specified limit i.e. 90–110%

**Table 2:** Physicochemical characterization of lornoxicam gels F1-F9 (n = 3).

Formulation Code	pH	Drug Content	Viscosity
		(%)	(cps)
F1	6.75 ± 0.07	100.46 ± 0.451	1300 ± 0.83
F2	6.25 ± 0.03	101 ± 0.152	5130 ± 0.59
F3	6.30 ± 0.04	100.2 ± 0.500	2850 ± 0.77
F4	6.55 ± 0.06	100.5 ± 0.65	3150 ± 0.34
F5	6.28 ± 0.03	98.45 ± 0.89	2250 ± 0.52
F6	6.42 ± 0.05	100.2 ± 0.79	3300 ± 0.75
F7	6.80 ±0.045	96.75 ± 0.34	2500 ± 0.64
F8	6.41 ± 0.01	99.9 ± 0.46	2300 ± 0.53
F9	6.5 ± 0.05	100.45 ± 0.59	1750 ± 0.82

### 3.3 Characterization of fabricated patches

The patches having a surface area of 30 cm^2^ were developed and evaluated for weight, thickness, and content uniformity. The mean weight of reservoir patches F1-F9 was found to be 5.325 ± 0.008 g– 5.702 ± 0.105 g and thickness between 1.32 ± 0.005 mm– 1.435 ± 0.004 mm. The results indicate that there was a slight difference in the weight and thickness among the formulations. Content uniformity between 99.05 ± 0.38% and 100.54 ± 0.12% indicated good uniformity of drug content among all the formulations.

### 3.4 In vitro release study

**Fig 1:**
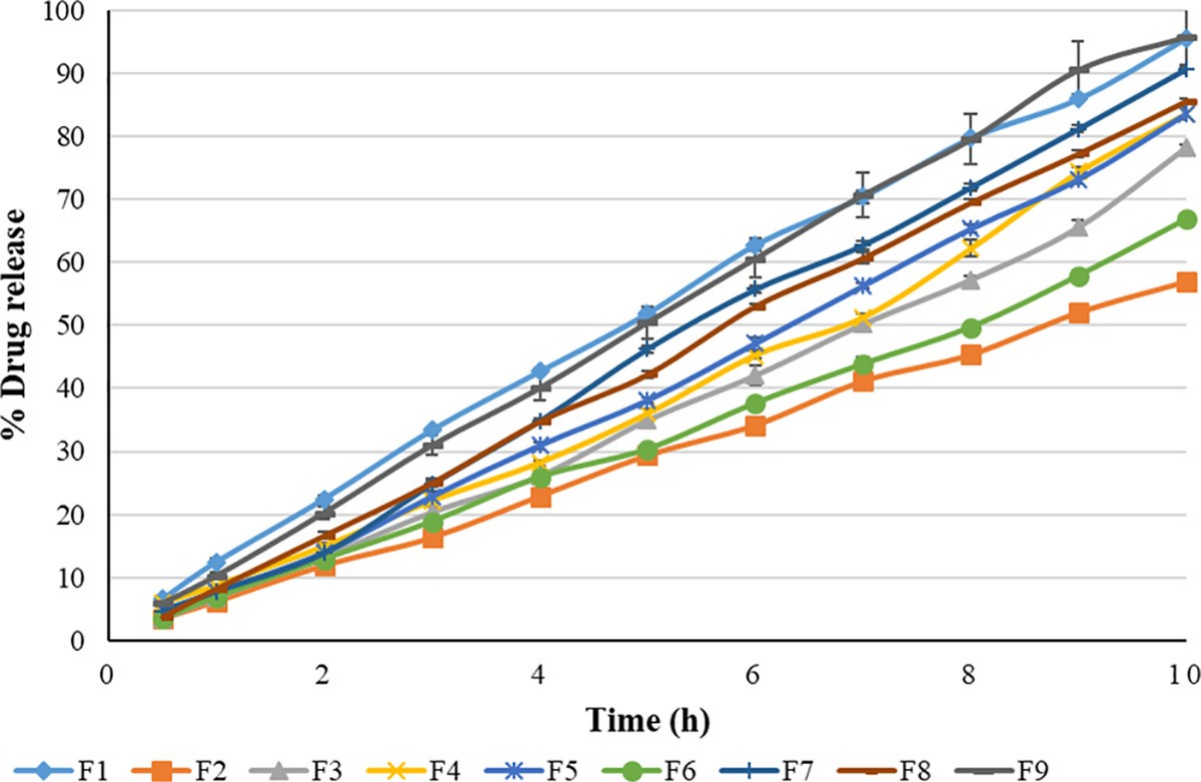
In vitro release profile of formulation F1-F9 (n = 3; mean ± SD) from lornoxicam reservoir patches.

An in vitro drug release evaluation experiment can give a reliable indication of the rate and extent of drug release from a transdermal patch. In reservoir-type transdermal patches, drug delivery is mainly governed by the release of drug from the patches.[40]. In such systems, there is an inherent secondary control due to its rate controlling membrane. Fig 1 represents the release profile which indicates maximum release from formulation F9 (95.8 ± 1.121%) followed by formulation F1 (95.63 ± 0.08%) in 10 h while formulations F2, F3 and F6 exhibited a release of less than 80%.

To study the release kinetics, data obtained from in-vitro drug release studies were plotted in various kinetic models which include zero order, first order, Higuchi, Korsmeyer-Peppas, Weibull and Makoid–Banakar. All the formulations best comply with Makoid–Banakar model showing R^2^ values in the range of 0.998–0.999 as shown in Table 3. The reservoir patches of estradiol [22] and dexibuprofen[41]. have also followed the Makoid-Banakar model. When the ‘c’ value of the Makoid-Banakar model approaches zero, the model becomes identical to the Korsmeyer-Peppas model. [42]. The release exponent ‘n’ indicates the type of diffusion followed by the formulation. In the current study, n values were found between 0.5–1 showing Non-Fickian transport. [23]

**Table 3:** Model fitting of the lornoxicam reservoir patches (F1-F9) release profile.

Mathematical models	F1	F2	F3	F4	F5	F6	F7	F8	F9
**Zero-order**									
R^2^	0.996	0.992	0.989	0.987	0.991	0.994	**0.995**	0.99	0.998
k_0_ (h^-1^)	9.861	5.712	7.24	7.88	7.971	6.377	8.827	8.372	9.905
**First-order**									
R^2^	0.947	0.967	0.928	0.9148	0.924	0.958	0.906	0.911	0.93
k_1_ (h^-1^)	0.167	0.074	0.101	0.114	0.116	0.086	0.135	0.124	0.167
**Higuchi model**									
R^2^	0.877	0.813	0.794	0.7969	0.807	0.822	0.796	0.839	0.892
k_H_ (h^-1/2^)	25.608	14.58	18.5	20.17	20.43	16.39	22.54	7.52	9.008
**Korsmeyer-Peppas model**									
R^2^	0.995	0.997	0.996	0.9938	0.995	0.995	0.991	0.997	0.996
K_kp_ (h^-n^)	12.198	4.9	5.369	5.985	6.471	5.717	6.729	6.25	10.793
n	0.894	0.774	0.938	0.836	0.873	0.954	0.934	0.945	0.959
**Weibull**									
R^2^	0.985	0.997	0.984	0.975	0.98	0.987	0.989	0.99	0.986
T_d_ (h)	5.888	11.398	8.582	7.842	7.752	10.146	6.879	7.283	5.903
Α	12.673	26.317	27.931	26.992	24.385	22.456	29.333	30.489	16.271
Β	1.432	1.344	1.549	1.6	1.56	1.343	1.752	1.721	1.571
**Makoid Banakar**									
R^2^	**0.999**	**0.998**	**0.999**	**0.999**	**0.998**	**0.998**	**0.998**	**0.997**	**0.999**
n	0.941	1.256	0.833	0.689	0.804	0.789	1.209	1.172	1.056
k_MB_	11.86	4.299	6.696	8.184	7.949	6.801	6.37	6.124	10.132
c	0.008	0.03	-0.053	-0.075	-0.05	-0.046	0.012	0.004	0.017

### 3.5 In vitro permeation study

**Fig 2:**
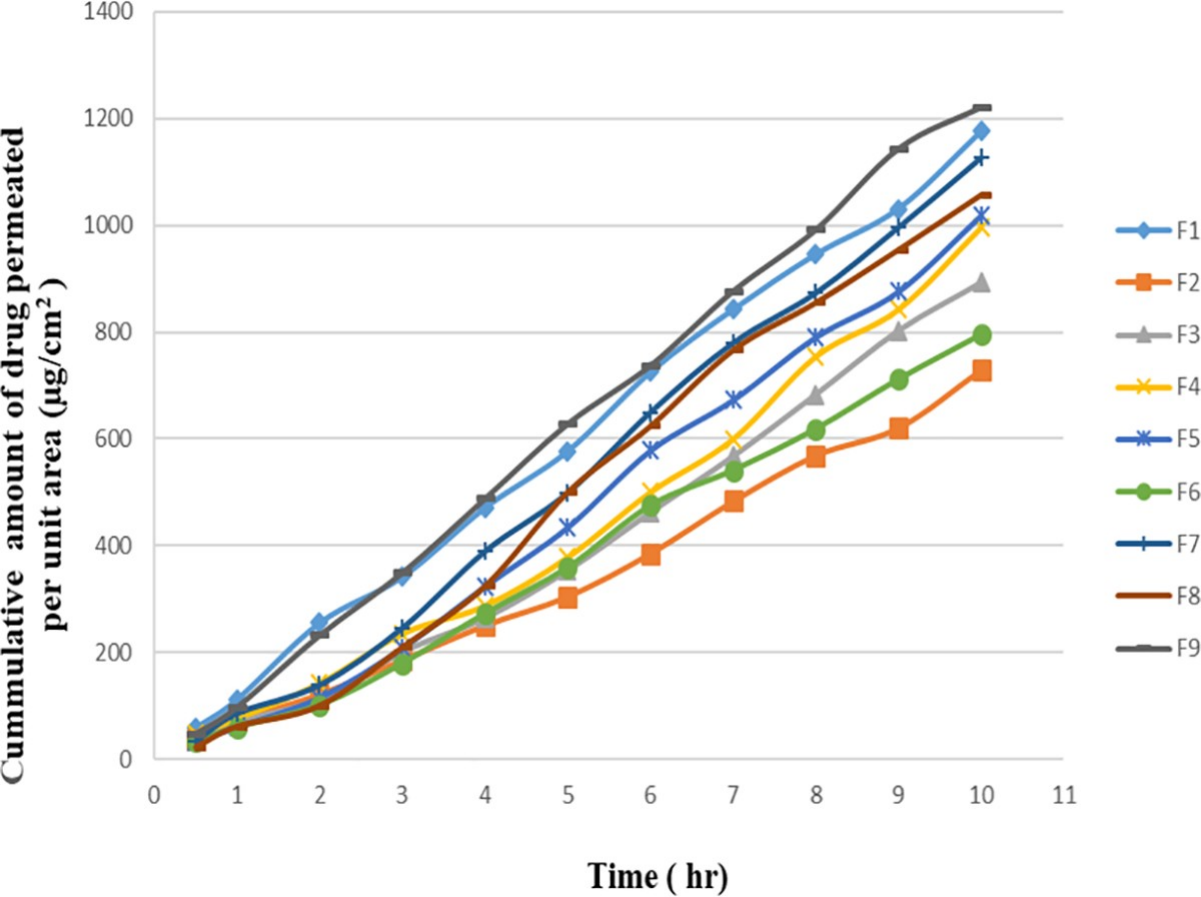
In vitro permeation profile of formulation F1-F9 (n = 3; mean ± SD) from lornoxicam reservoir patches.

The permeation studies were carried out using whole rat skin. Full thickness abdominal skin was excised from Wistar albino rats and hair of the rats was removed with a clipper. Subcutaneous tissues, fats and tissues were also removed. The skin samples were cut into appropriate size for permeation studies. [7]. Fig 2 represents the permeation profile of formulated reservoir patches. The cumulative amount of LRX permeated per unit area from F1 and F9 was found to be 1179.2±6.87 μg/cm^2^ and 1217.77±1.16 μg/cm^2^, respectively. The permeation parameters were computed and presented in Table 4.

**Table 4:** Permeation parameters of lornoxicam reservoir patches (F1-F9).

Formulation code	Flux	Lag time	Permeability coefficient	Diffusion coefficient	F_D_	F_S_	Regression coefficient	Best fit equation for permeation plot
	(J)	(t_lag_)	(P)	(D)				
	μg/cm^2^/h	H	cm/h	cm^2^/h				
F1	122.52±1.28	0.935±0.29	3.06E-03	5.26E-05	0.923	0.077	0.9961	Q = 122.52t – 1.225
F2	67.74±1.14	1.692 ± 0.09	1.69E-03	2.91E-05	0.911	0.089	0.9923	Q = 67.74t – 6.488
F3	93.56±1.22	1.225± 0.24	2.33E-03	4.02E-05	0.862	0.138	0.9887	Q = 93.56t – 63.063
F4	101.50±0.78	1.129± 0.26	2.53E-03	4.36E-05	0.905	0.095	0.9822	Q = 101.5t – 70.16
F5	106.29±0.53	1.078 ± 0.45	2.65E-03	4.57E-05	0.929	0.07	0.9821	Q = 106.3t –76.403
F6	80.28±1.23	1.428±0.38	2.00E-03	3.45E-05	0.888	0.112	0.9886	Q = 80.28t – 27.55
F7	117.42±0.75	0.978±0.12	2.93E-03	5.05E-05	0.973	0.027	0.9921	Q = 117.42t – 69.89
F8	115.98±1.27	0.958 ±0.43	2.89E-03	4.98E-05	0.914	0.086	0.988	Q = 115.99t – 94.20
F9	126.51±1.19	0.908 ± 0.57	3.16E-03	5.43E-05	0.955	0.045	0.9986	Q = 126.51t – 22.768

Almost 91.32% of the drug was permeated from formulation F9 in 10h with a maximum flux of 126.51±1.19 μg/cm^2^/h and a minimum lag time of 0.908 ± 0.57h whereas, the permeability coefficient was found to be 3.16E-03 cm/h. In a study conducted by Yener et al, the permeation coefficient of the LRX transdermal patch was found to be 1.34E-3 cm/h in the presence of single penetration enhancer[43]. In another study, when OA and PG were used separately, the flux of LRX transdermal patches was found to be 17.97 ± 0.463 μg/cm^2^/h and 39.45 ± 0.74 μg/cm^2^/h, respectively.[44]. The results of the present study reveal that the presence of permeation enhancers as a cosolvent produces a significant impact on the permeation of LRX across the membrane. Similar results have been reported in previous studies where the combination of fatty acids and PG has produced significant enhancement on skin permeation rate of nicardipine, [45] physostigmine[46] and ketoprofen.[47].

Cosolvents have been widely used as vehicles as well as penetration enhancers in the transdermal formulation of drugs. In addition to affecting the drug solubility in the vehicle, cosolvents may alter the structure of the skin and modify the penetration rate. Thus, cosolvents can affect both drug release and percutaneous absorption. Moreover, the use of a cosolvent may offer synergistic enhancement. [12]

It has been revealed that there are separate hydrophilic and lipophilic domains present in the barrier area of the stratum corneum. Therefore, penetrants exhibiting both hydrophilic and lipophilic properties can probably penetrate stratum corneum more readily. Fatty acids are known to be enhancers with lipophilic properties and various studies have shown that the skin permeability enhancing effects of fatty acids are greater with PG. This binary system can disorganize the multilaminate hydrophilic-lipophilic layers located intercellularly in the stratum corneum, consequently promoting percutaneous absorption of drugs [48]

In the current investigation, it was observed that the presence of PG and OA has shown a good correlation between drug release and permeation of LRX. Maximum drug release (95%) and permeation (91.32%) can be achieved with this binary system of penetration enhancers. The drug release profile indicates a controlled release of LRX for 10h with a rate that is almost similar to that of the drug delivery rate through the rat skin. The Fd and Fs values were also calculated as shown in Table 4. The Fd values ranges from 0.862–0.955 for formulations F1-F9. Kalia and Guy [31] suggested that the value of Fd closer to 1 indicates that the device majorly controls drug permeation.

### 3.6 Effect of formulation factors

**Fig 3:**
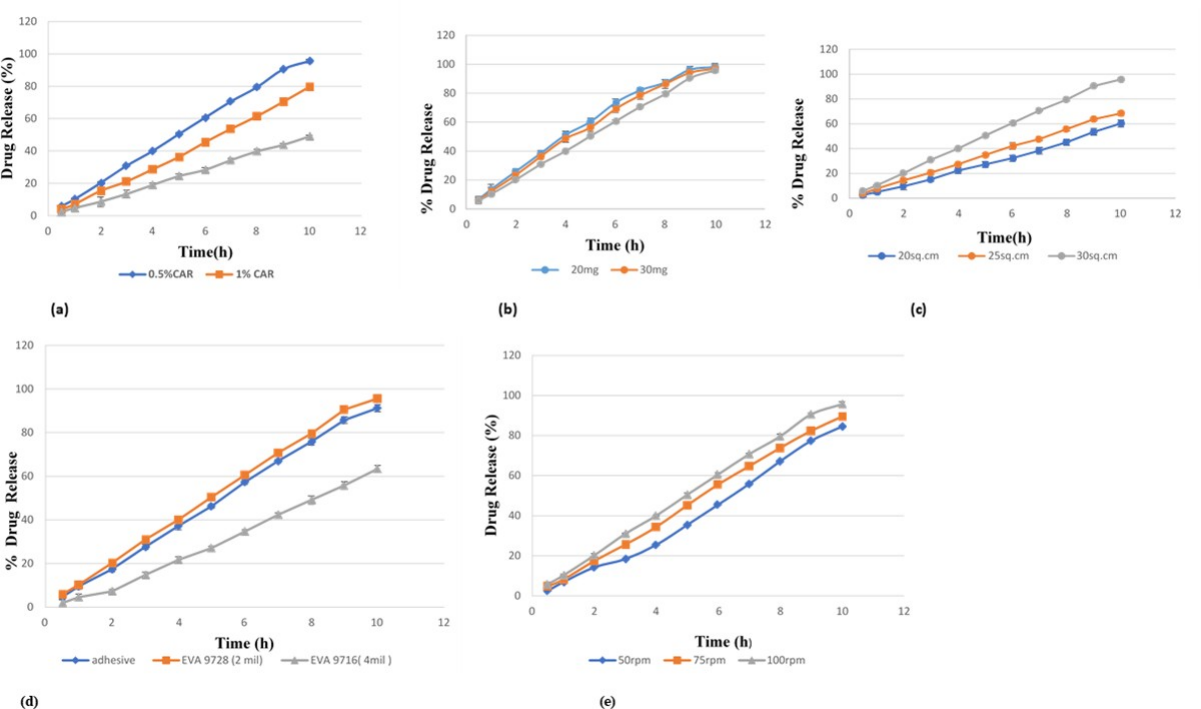
**(a):** Effect of gelling agent concentration on drug release. **(b):** Effect of drug loading on drug release. **(c):** Effect of surface area on drug release. **(d):** Effect of transdermal components on drug release. **(e):** Effect of agitation speed on drug release.

**Fig 4:**
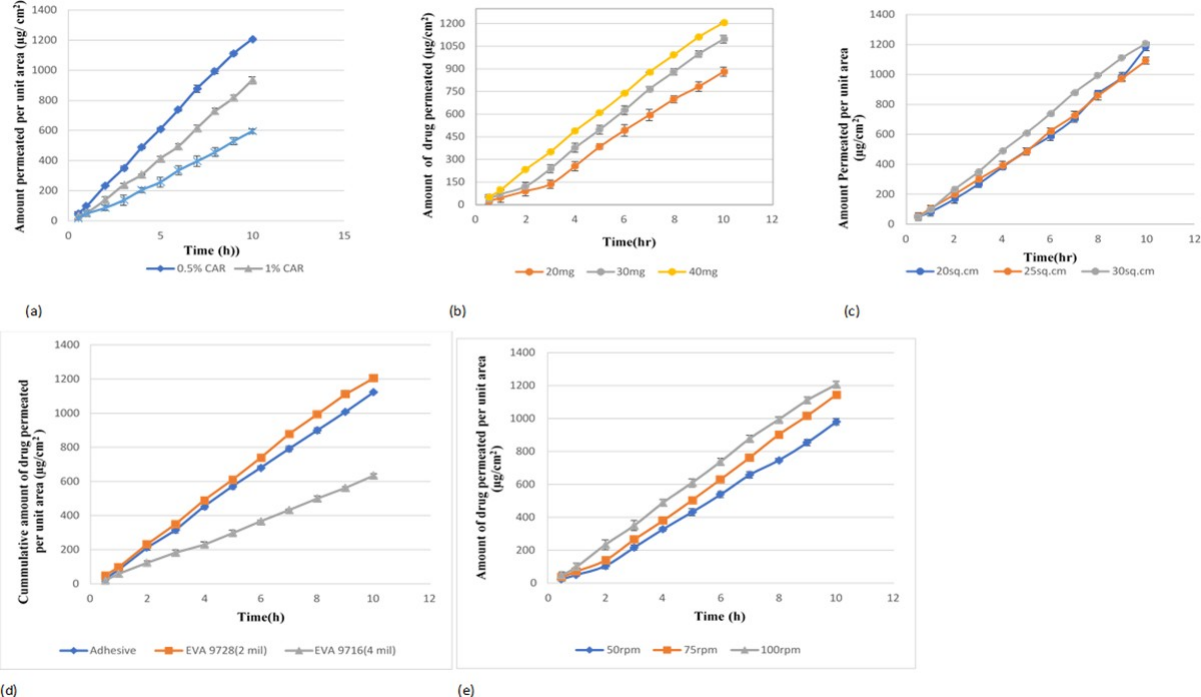
**(a):** Effect of gelling agent concentration on drug permeation. **(b):** Effect of drug loading on drug permeation. **(c):** Effect of surface area on drug permeation. **(d):** Effect of transdermal components on drug permeation. **(e):** Effect of agitation speed on drug permeation.

Different formulation factors such as gelling agent concentration, drug loading, surface area and rate controlling membrane were studied for drug release (Fig 3(A), 3(B), 3(C) and 3(D)) and drug permeation characteristics (Fig 4(A), 4(B), 4(C) and 4D)).

#### 3.6.1 Effect of gelling agent concentration

The impact of varying concentration of gelling agent (0.5–1.5%) on release and permeation was investigated. It was observed that the increase in carbopol concentration has decreased the drug release and the rate of permeation across the skin as presented in Figs 3(A) and 4(A), respectively. This is similar to the findings of Patel et al. where an increase in the concentration of gelling agents leads to a significant decrease in drug release. [49]. This may be attributed to the increase in microviscosity of gel leading to a decrease in the drug release and permeation. **[50]** The complex gel network has caused a longer diffusion pathway of the drug to be permeated through the membrane.

#### 3.6.2 Effect of drug loading

The drug loading effect was evaluated by formulating patches containing varying quantities of LRX (20 mg, 30 mg, and 40 mg) and is presented in Figs 3(B) and 4(B). Lower drug loading leads to a faster release of the drug due to the formation of the drug enriched shell. [51] It was also observed in the current study, that the higher drug loading has significantly reduced the release of drug from patches. Whereas, flux has increased from 95.75μg/cm^2^/h to 126.51 μg/cm^2^/h from the patches containing 20 mg and 40 mg drug, respectively. Similar results were reported in a study where high skin permeation of benztropine was obtained with a higher drug loading in patch formulations. According to Fick’s Law, skin permeation of a drug is usually proportional to the drug concentration when the drug concentration is below saturation. [52] There was good linearity between the skin permeation and the loading amount of Lornoxicam.

#### 3.6.3 Effect of surface area

The surface area of the patch in contact with skin is the predictor of drug release. Patches of the variable surface area (20 cm^2^, 25 cm^2^ and 30 cm^2^) were also fabricated to evaluate the effect of surface area on drug release and permeation. Drug release at 20 cm^2^, 25 cm^2^ and 30 cm^2^ was found to be 60.27%, 68.41% and 95.8%, respectively. It was observed that drug release was dependent on the area of the devices as shown in Fig 3(C). When the diameter of the device was increased, drug release was also increased. Thacharodi et al also concluded that the surface area of device affect the drug release and decrease in the area has also reduced the release of drug from the device [53]

Fig 4(C) represents drug permeation from LRX patches with different surface area and was found approximately similar i.e 115.58 μg/cm^2^/h, 110 μg/cm^2^/h and 126.51 μg/cm^2^/h, respectively

Similar findings were reported by Ali et al, where a change in the surface area resulted in a different release profile but approximately similar permeation profile.[41].

#### 3.6.4 Effect of rate-controlling membrane

The impact of rate-controlling membrane on drug release and permeation was also examined by using EVA membranes having variable vinyl acetate content i.e. 19% (3M CoTran-9728) and 9% (3M CoTran-9716). It was observed that the drug release and flux has increased with the increase in vinyl acetate content as shown in Figs 3(D) and 4(D). This may be attributed to the difference in vinyl acetate content in EVA membranes. These results were in accordance with Shin et al where the increase in vinyl acetate content resulted in increased drug release and permeation. [54]. Yang et al also reported the use of EVA membrane with 19% vinyl acetate content as a suitable controlled-release membrane for the development of transdermal reservoir patch of bufalin. [55]. Therefore, EVA membrane 9728 was selected for designing the optimized formulation.

#### 3.6.5 Effect of adhesive

The selection of suitable pressure-sensitive adhesive is an important aspect of developing the transdermal delivery system[56]. Besides the drug itself, PSAs can also affect drug delivery from the developed patch. Therefore, the selection of an appropriate PSA is an important factor in designing a transdermal delivery system. In the current study, the effect of adhesive on drug release and permeation was evaluated using Duro-Tak 387/2510. The presence of carboxyl functional group in this adhesive contributes significantly to increase solubility and drug release[14] It has been reported that the use of Duro-Tak 387/2510 increases the permeation more as compared to other adhesives[57]. Figs 3(D) and 4(D) presents the release and permeation profile of membrane coated with adhesive and without adhesive. The drug release and permeation with adhesive was 91.24 ± 1.2% and 1122.59 ± 4.13 μg/cm^2^ while without adhesive, the release and permeation were observed as 95.8 ± 1.18% and 1217.77 ± 1.16 μg/cm^2^, respectively.

#### 3.6.6 Effect of agitation speed

Figs 3(E) and 4(E) represent the impact of agitation speed on release and permeation. Minor variations in stirring rates don’t impact the release profile significantly [58]. This was also observed in the current study where variation in agitation speed lead to a slight difference in the release and permeation profiles. The release from lornoxicam patches agitated at 50 rpm, 75 rpm, and 100 rpm was found to be 84.5 ± 0.46%, 89.46 ± 0.55% and 95.8 ± 1.12%, respectively and flux was found to be 102.97 μg/cm^2^/h, 119 μg/cm^2^/h and 126.51 μg/cm^2^/h, respectively.

### 3.7 Formulation optimization

3^2^ Full factorial design was selected for the optimization of LRX patches. Two factors were evaluated each at 3 levels and experimental trials were carried out at all 9 possible combinations. PG (*X*_1_) and OA (*X*_2_) were selected as independent variables whereas dependent variables were Q_10_ (*Y*_1_), flux (*Y*_2_) and lag time (*Y*_3_). A statistical model incorporating interactive and polynomial terms was used to evaluate the responses.

$$Y=b_{0}+b_{1}X_{1}+b_{2}X_{2}+b_{12}X_{1}X_{2}+b_{11}X_{1}^{2}+b_{22}X_{2}^{2}$$ (14)

Where *Y* represents the dependent variable, *b*_0_ is the arithmetic mean response of the 9 runs, and *b*_*i*_ is the estimated coefficient for the factor *X*_*i*_. The effects (*X*_1_) and (*X*_2_) indicate the average result of changing 1 factor at a time from its low to high value. The interaction terms (*X*_1_*X*_2_) describes the change in response when 2 factors are changed simultaneously. The polynomial terms ($X_{1}^{2}$) and ($X_{2}^{2}$) are added to observe nonlinearity. Data analysis was performed using Design-Expert 11 software (Stat ease, Minneapolis, MN)

The results clearly indicate that the drug release at 10^th^ h, flux and lag time were strongly dependent on the selected independent variables. The quadratic model was observed as the best-fitted model. Insignificant terms with P>0.05 were removed to generate reduced models. However, the terms having P<0.05 were considered statistically significant and were retained in the reduced models. Equations were developed for reduced quadratic models of *Y*_1_ (Q_10_), *Y*_2_ (flux) and *Y*_3_ (lag time) which are given as:

$$Y_{1}=90.06+8.67A–8.51B–12.19A^{2}$$ (15)

$$Y_{2}=116.74+13.76A–12.31B–19.28A^{2}$$ (16)

$$Y_{3}=0.99–0.19A+0.16B+0.25A^{2}$$ (17)

The predicted values of formulations were also generated, Table 5 represents the comparative levels of experimental and predicted responses of different lornoxicam reservoir patches which suggests that the predicted values for Q_10_ (*Y*_1_), flux (Y_2_) and lag time (Y_3_) were very close to that of experimental values. Table 6 describes the summary statistics for reduced quadratic models. The predicted R^2^ values for responses *Y*_1_,*Y*_2_ and *Y*_3_ are in reasonable agreement with the adjusted R^2^.

**Table 5:** Comparison of experimental results (mean ± SD; 𝑛 = 3) and predicted values.

Formulation code	Experimental responses			Predicted Responses		
	Q_10_ (%)	J (μg/cm^2^/h)	t_lag_ (h)	Q_10_ (%)	J (μg/cm^2^/h)	t_lag_ (h)
F1	95.63±1.12	122.52± 1.28	0.935±0.29	95.51	123.53	0.89
F2	57.01±0.40	67.74± 1.14	1.692±0.09	60.66	71.39	1.59
F3	78.34±0.53	93.56± 1.22	1.225±0.24	78.06	96.68	1.21
F4	83.69±0.83	101.50± 0.78	1.129±0.26	82.74	98.01	1.19
F5	83.66±0.96	106.29± 0.53	1.078 ± 0.45	81.47	104.43	1.15
F6	66.91±0.66	80.28 ± 1.23	1.428± 0.38	69.2	83.7	1.45
F7	90.73±0.62	117.42± 0.75	0.978± 0.12	91.55	118.19	0.93
F8	85.65±0.38	115.98± 1.27	0.958 ± 0.43	86.6	112.22	1.05
F9	95.8±0.08	126.51± 1.19	0.908 ± 0.57	95.54	121.73	0.93

**Table 6:** Model summary statistics (for reduced quadratic model).

Responses	PRESS	R^2^	Adjusted R^2^	Predicted R^2^	Adequate precision	SD	%CV	F-value	P-value	Comments
									Prob>F	
Y_1_	543.37	0.987	0.9845	0.9843	10.253	5.56	6.55	12.85	0.0086	significant
Y_2_	927.66	0.9042	0.9038	0.9035	11.317	7.35	7.05	15.52	0.0054	significant
Y_3_	0.16	0.981	0.9095	0.9093	9.89	0.092	8.47	12.25	0.0099	significant

Values of “Prob > F” less than 0.0500 indicate model terms are significant. Values greater than 0.1000 indicate the model terms are not significant. Model reduction has been done to improve the model.

**Fig 5:**
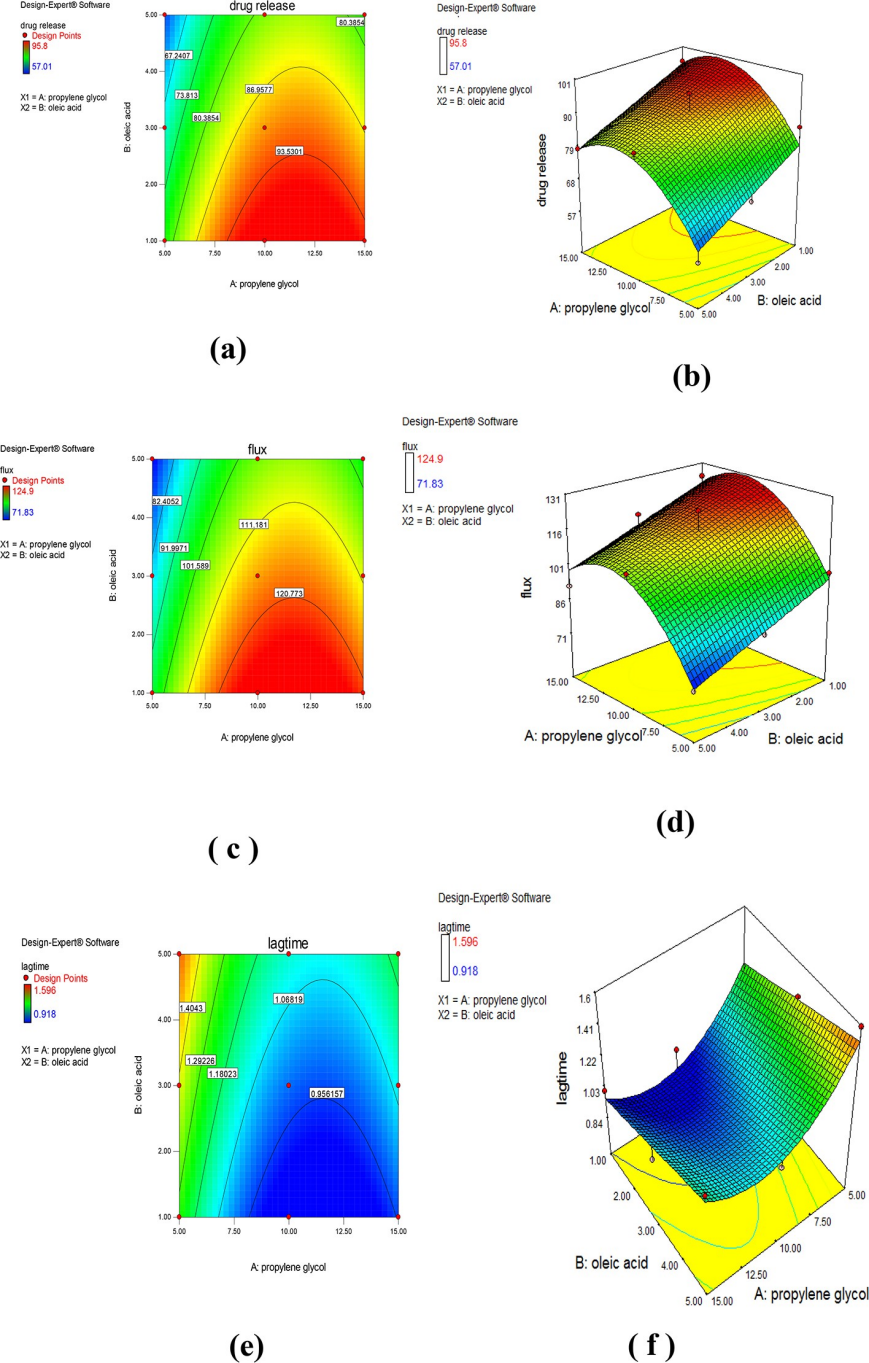
Contour plots **(a)** 𝑄_10_ **(c)** Flux **(e)** Lag-time and responses surface curves **(b)** 𝑄_10_ **(d)** Flux **(f)** Lag-time for optimization of lornoxicam reservoir patches.

The formulation F9 consisting of 0.5% carbopol, 10% PG and 3% OA was considered as the optimized formulation of LRX reservoir patch with Q_10_ (95.8%), flux (124.9 μg/cm ^2^/h) and lag time (0.918 h). Fig 5 represents contour plots and 3D response surface plots indicating that the maximum release, flux and minimum lag time were observed when mid-value of PG and OA were used.

### 3.8 Skin irritation study

Transdermal systems are intended for application on the skin, hence it is necessary to examine the biocompatibility of such formulations with the skin. The pressure sensitive adhesives used for adhering the patch may produce skin reactions. Therefore, skin irritation study is essential to examine the skin sensitivity to the applied patches[59]. The results obtained for skin irritation study showed satisfactory results as shown in Table 7. According to Draize et al, compounds that produce scores of 2 or less are considered negative i.e. no skin irritation. [32]. Hence, the fabricated LRX patch was declared safe for use.

**Table 7:** Skin irritation study of optimized lornoxicam reservoir patch (mean ± SD; 𝑛 = 6).

Rat No.	Control		F9		Formalin	
	Erythema^a^	Edema^b^	Erythema^a^	Edema^b^	Erythema^a^	Edema^b^
1	0	0	1	0	3	2
2	0	0	2	1	2	3
3	0	0	0	1	3	1
4	0	0	1	0	2	2
5	0	0	2	2	2	3
6	0	0	1	0	2	2
Average	0	0	1.16 ± 0.752**	0.66 ± 0.816**	2.333 ± 0.516	2.166 ± 0.752

**p<0.01, significant when compared to formalin

a: Erythema scale: score 0, no erythema; score 1: very slight erythema; score 2: well defined erythema; score 3: moderate to severe erythema; and score 4: severe erythema and scar formation.

b: Edema scale: score 0: no edema; score 1: very slight edema; score 2: slight edema; score 3: moderate edema; and score 4: severe edema

### 3.9 In vivo analgesic and anti-inflammatory activities

#### 3.9.1 Hot-plate test

In order to evaluate the analgesic efficacy of the formulated LRX reservoir patches, Hot-plate studies were carried out using Wister albino rats. The analgesic activity of Lornoxicam patch was manifested by their resistance or tolerability to the sensation of heat until licking their paws or jumping. Findings from this study demonstrated that the newly formulated LRX patch exhibited significant analgesic effect against thermal pain stimuli as shown in Table 8. Similar results were demonstrated by Baviskar et al where the matrix-type lornoxicam patches exhibited potent analgesic effect against thermal pain stimuli.[35]. Statistically, a significant difference was observed (P < 0.01) in the reaction time between the test and control group. However, the difference between the reaction time of the test and the standard group was found to be insignificant (P > 0.05).

**Table 8:** Analgesic effect of optimized lornoxicam reservoir patch on the reaction time of rats by hot plate method (mean ± SD; 𝑛 = 6).

Group	Treatment	Reaction time in seconds									
		Paw licking					Jump Response				
		0 min	30 min	60 min	120 min	180 min	0 min	30 min	60 min	120 min	180 min
I	Control	35.5 ± 0.4	38.2 ± 0.3	38.8 ± 0.6	36.3 ± 0.5	38.4 ± 0.4	74.2 ± 0.8	75.4 ± 1.2	75.8 ± 0.9	74.3 ± 1.1	75.2 ± 0.5
II	Standard	37.5 ± 0.5	43.4 ± 0.7**	50.2 ± 0.4**	55.4 ± 0.7**	60 ± 0.5**	76.3 ± 0.7	82 ± 0.6**	88.4 ± 0.4**	95 ± 0.6**	103 ± 0.9**
III	Test	36.3 ± 0.24	44.5 ± 0.6**	49.22 ± 0.52**	53.35 ± 0.25**	58.88 ± 0.74**	74.35 ± 0.55	84.4 ± 0.56**	89.5 ± 0.3**	94 ± 0.4**	101 ± 0.6**

**P<0.01, significantly different from control (Tukey’s multiple comparison post–hoc test)

#### 3.9.2 Acetic acid induced writhing response

Acetic acid-induced writhing method was also adopted for the evaluation of the analgesic activity. Writhing is defined as a stretch, tension to one side, extension of hind legs, contraction of the abdomen so that the abdomen of mice touches the floor, turning of the trunk (twist). Any writhing is considered as a positive response[60] In order to assess the analgesic effect of the drug, it is considered as highly sensitive and useful method. The current study reveals that the test and standard drug significantly (P < 0.01) reduces the number of abdominal constrictions and stretching of hind limb induce by the injection of Acetic acid as presented in Table 9. The selected formulation was also compared to the standard and the difference between them was found to be insignificant (P>0.05).

**Table 9:** Effect of optimized lornoxicam formulation on 1% acetic acid induced writhes in the rat model (mean ± SD; 𝑛 = 6).

Group	Treatment	Writhes (n)	%age inhibition of Writhes
I	Control	16 ± 0.57	-
II	Standard	8.5 ± 0.28**	46.87
III	Test	10 ± 0.15**	37.5

**P<0.01 significantly different from control (Tukey’s multiple comparison post -hoc test).

In general acetic acid causes pain by liberating endogenous substances such as serotonin histamine, prostaglandins, bradykinins.[61] The significant increase in pain threshold by test and standard in these models suggests inhibition of PGs, Leukotrienes and other endogenous substances which are mainly responsible for producing pain.

#### 3.9.3 Carrageenan hind-paw edema test

Carrageenan-induced rat paw edema is a commonly used model for studying the anti-inflammatory effect of drugs in rats. Carrageenan induces inflammation that is acute, non-immune and reproducible, which is characterized by an increase in the size of hind paw[62].

**Fig 6:**
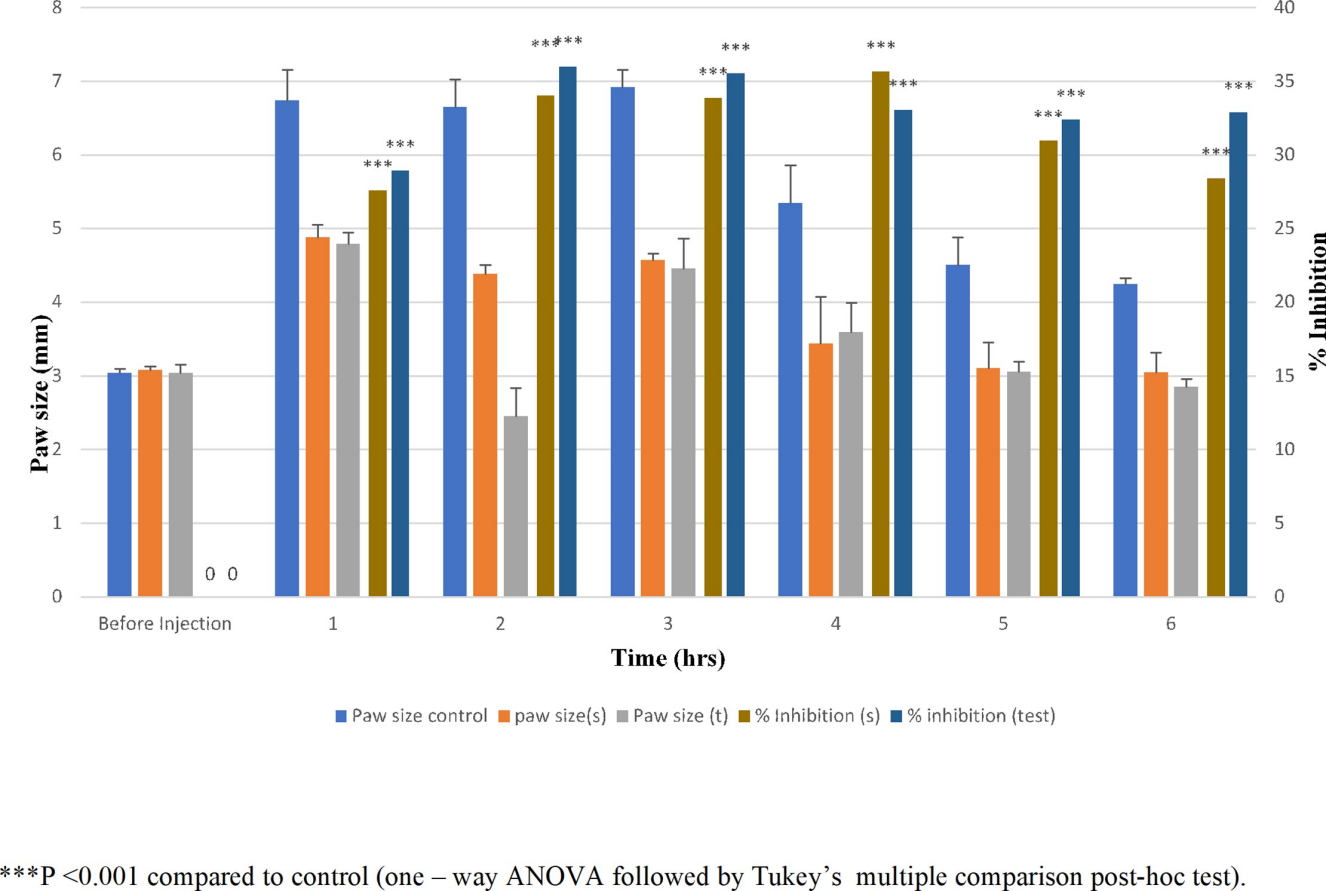
Graphical presentation of anti-inflammatory activity of optimized lornoxicam reservoir patch; swelling of hind paw of control, standard and test rats (n = 6; mean ± SD) and % inhibition of standard and test.

In the present investigation, the swelling in the paw size of rats was observed for 6h and % inhibition was calculated. As shown in Fig 6, there is a significant difference (P<0.001) in the swelling of paw size between control and test group. However, no significant difference (P>0.05) was observed between orally administered LRX and test group. The percent inhibition of edema is also represented graphically indicating a significant reduction in hind paw swelling after application of test patch followed by orally administered lornoxicam. These results were in good agreement with Nabarawi et al where both oral and transdermal groups showed increased inhibition than the control group.[44]

Smith et al [63] reported that NSAIDs mainly produce their effect by inhibition of COX enzymes. COX exists in two isomeric forms as COX 1and COX 2. LRX was found to be a potent inhibitor of COX1 and COX 2 and produce marked analgesic and anti-inflammatory effects.

LX is at least 10 times more potent as an antiinflammatory agent than piroxicam and was also tenfold more active than tenoxicam in inhibiting carrageenan-induced edema in the rat paw swelling in the adjuvant-induced polyarthritic rat [64]

### 3.10 Stability studies

**Fig 7:**
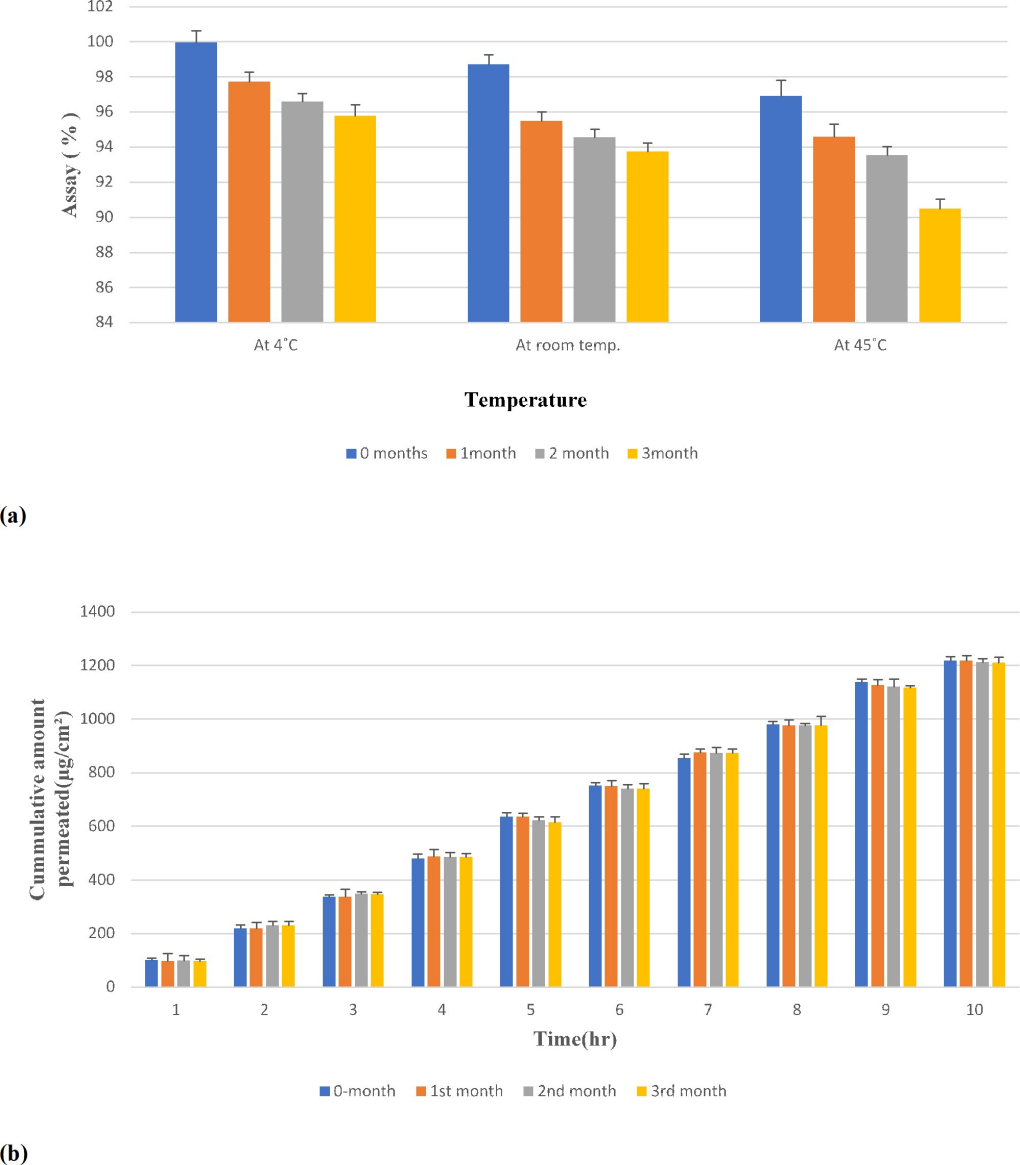
**(a):** Stability profile of lornoxicam reservoir patch at different temperatures. **(b):** Stability comparison profile of permeation (0–3 months) of lornoxicam patch at 4˚C.

The stability testing of LRX patch was carried out at 4˚C, room temperature, and 45˚C over a period of 3 months. Fig 7(A) represents the LRX content in the patch after 3 months, which was found to be 95.79 ± 1.80%, 93.75 ± 2.24% and 90.50 ± 1.55% when kept at 4˚C, room temperature, and 45˚C, respectively. It was observed that drug content was most affected at a higher temperature. Though potency loses at a higher temperature, no other interference was observed for other components in the patch. There was no extra peak in the chromatogram. Similar results had been reported by Kusum et al. [65]

The shelf-life for LRX patches was also calculated and was found to be 196 days at 4˚C, 140 days at room temperature and 131 days at 45˚C. Thus, it is suggested to store the patches in the refrigerator due to their prolonged shelf life at low temperatures.

In reservoir systems, the absorption of permeation enhancers by polymers of the membrane may lead to failure in the permeation of drugs across the skin.[66] The integrity of penetration enhancers was evaluated through the determination of transdermal permeation across rat skin. The results presented in Fig 7(B) show that drug permeation did not alter after three months when patches were stored at 4˚C indicating the persistence in the integrity of penetration enhancers.

## Conclusions

The membrane-based transdermal patches of LRX gel exhibiting controlled release was formulated. The use of OA and PG co-solvent as penetration enhancer were found effective in improving the flux of drug through the skin. The developed reservoir patches resulted in suitable analgesic and anti-inflammatory activity with no observed skin irritation. Thus, by achieving an effective therapeutic level of LRX with improved patient compliance, reduced gastrointestinal side-effects and dosing frequency, these transdermal patches can be used as an alternative to oral administration of Lornoxicam.

## Supporting information

S1 Data (ZIP)
